# Detection of Acidic Pharmaceutical Compounds Using Virus-Based Molecularly Imprinted Polymers

**DOI:** 10.3390/polym10090974

**Published:** 2018-09-01

**Authors:** In-Hyuk Baek, Hyung-Seop Han, Seungyun Baik, Volkhard Helms, Youngjun Kim

**Affiliations:** 1 Environmental Safety Group, Korea Institute of Science & Technology Europe GmbH, 66123 Saarbrücken, Germany; ih.baek@kist-europe.de (I.-H.B.); sbaik@kist-europe.de (S.B.); 2 Center for Bioinformatics, Saarland University, 66123 Saarbrücken, Germany; volkhard.helms@bioinformatik.uni-saarland.de; 3 Nuffield Department of Orthopaedics, Rheumatology and Musculoskeletal Sciences, University of Oxford, B4495 Oxford, UK; hyungseop.han@ndorms.ox.ac.uk

**Keywords:** molecularly imprinted polymer, polypyrrole, filamentous bacteriophage, clofibric acid, self-assembly, virus, electrochemical polymerization, microbalance sensor

## Abstract

Molecularly imprinted polymers (MIPs) have proven to be particularly effective chemical probes for the molecular recognition of proteins, DNA, and viruses. Here, we started from a filamentous bacteriophage to synthesize a multi-functionalized MIP for detecting the acidic pharmaceutic clofibric acid (CA) as a chemical pollutant. Adsorption and quartz crystal microbalance with dissipation monitoring experiments showed that the phage-functionalized MIP had a good binding affinity for CA, compared with the non-imprinted polymer and MIP. In addition, the reusability of the phage-functionalized MIP was demonstrated for at least five repeated cycles, without significant loss in the binding activity. The results indicate that the exposed amino acids of the phage, together with the polymer matrix, create functional binding cavities that provide higher affinity to acidic pharmaceutical compounds.

## 1. Introduction

The rapid increase in the human lifespan and advancements in pharmaceutical technology have significantly improved the quality of human life in modern society. However, parallel to this, the uncontrolled usage and excretion of pharmaceutical compounds have become one of the biggest environmental concerns, as these compounds are widely detected in the oceans, surface water, ground water, and soils [1]. Clofibric acid (CA) is one of the metabolite forms of prescribed fibrate drugs (e.g., etofylline clofibrate, etofibrate, and clofibrate). These are commonly used in prescription drugs to treat diseases related to lipid-lowering symptoms in the blood of both humans and domestic animals [2]. Following digestion, CA and bioactive metabolite products are excreted from the body through urine and feces. Considering their effects on the mammalian lipid metabolism, these pharmaceutical metabolites might interfere with the lipid homeostasis and growth of non-target exposed animals, such as fish, as recently shown by the regulation of the genes that code for fatty acid-binding proteins and the enzyme fatty acyl-coenzyme-A oxidase, which is involved in fatty acid oxidation [3,4].

Molecularly imprinted polymers (MIPs) are a central part of chemical extraction systems or biosensors, which depend on functionalized noncovalent recognition cavities. The MIPs fulfilled new demands, particularly in the environmental monitoring and selective purification of endocrine disruptors, acidic pharmaceuticals, chemicals, and other emerging pollutants in contaminated water [5,6,7,8]. MIPs can also be used in various fields such as antibody, receptor mimics for biosensor, separation for chromatography, and catalysis for chemical synthesis [8,9,10,11]. The recognition elements in biosensors usually consist of antibodies, enzymes, or other biological receptors that are immobilized on the sensor surface. Conducting polymers show metal-like behavior because of conjugated double bonds, for example, polyacetylene, polypyrrole, polythiophene, and polyaniline [12]. The polymerization can be conducted at room temperature in organic solvents or aqueous solutions. This is advantageous for the imprinting of biomolecules, because denaturation and conformational changes can be avoided [13]. Polypyrrole is one of the first routinely electrochemically synthesized polymers [14]. The biocomposites of conductive polymers and virus particles have been prepared with M13 filamentous bacteriophage, tobacco mosaic virus (TMV), and human infected viruses, and conductive polymers have been prepared by chemical polymerization [15,16,17,18]. MIPs could be advantageous for robustness and reusability, because the functional cavities in MIPs are resilient to the environmental conditions of temperature, pressure, and pH [19,20]. MIPs could be applied in various fields, including solid-phase, chemical sensors, chromatography, in situ cell biology, diagnosis, and immunoassay, owing to the separation and recognition of the target chemicals [21,22]. A quartz crystal microbalance with dissipation monitoring (QCM-D) functions as a transducer to monitor tiny changes in the mass of sensitive elements (e.g., antibody, aptamers, receptor proteins, and MIP) and to send the signal to the computers [23]. The QCM-D can make two types of measurement, which are frequency (∆f) and dissipation (∆D). ∆f is related to the changes in the binding mass and ∆D is related to the viscoelasticity [24]. Filamentous bacteriophages (M13, F1, f88, and fd) are members of the family, *Inoviridae*. The proportion of filamentous bacteriophage are covered with 2700 copies of major coat protein pIII (50 amino acid [aa]), and five copies of minor protein pVII (33 aa), pIX (32 aa), pVI (112 aa), and pIII (406 aa) [25]. The linear structures of bacteriophage are approximately 6.6 nm in diameter and 100 nm in length [26,27]. Five capsid proteins have been used for phage display libraries, which can be modified into the phage vector of interest protein sequence using genetic engineering technics. The filamentous bacteriophage vector can transform into *Escherichia coli* and replicate the bacteriophages, which produce a large number of virus titers of 1.0 × 10^13^ in 250 mL of culture [28]. Donavan et al. showed that composites of poly (3,4-ethylenedioxythiophene) and filamentous bacteriophage M13 can be prepared by electrodeposition [26]. Additionally, the development of bacteriophage-based biosensors has been described. These were used for the recognition of specific target molecules as well as biomarkers [29], including the detection of bacterial pathogens in food [30], processed wastewater [31], and toxic insecticide [32], as well as the isolation of pharmaceutical compounds from waste [6] (human or animal blood), urine, or sputum, for clinical diagnostics [33].

The bacteriophage-driven hybrid biocomposites fabricated by electrochemical polymerization include PEDOT-M13 [18], polyaniline-M13 [15], and polypyrrole-T7 [34]. Upon exposure to solutions containing a target molecule that binds to the virus particles, the films of the virus-PEDOT biocomposite showed increased electrical impedance, and the nanowires showed increased DC resistance [18]. Filamentous bacteriophages (M13, f1, f88, and fd) have been used for various applications in material science [35], drug delivery [36], imaging [37], tissue engineering [38], energy storage [39], and as biosensors [18].

It has been demonstrated in many cases that MIP adsorption is an effective and economical method to sense and treat many pollutants in wastewater [40]. In contrast to this, CA separation using MIP [19] or the MIP adsorbent has drawn little attention. Furthermore, the MIP mentioned above was prepared using a single template, and thus these MIPs could not exhibit a high selectivity for most compounds, especially those belonging to different groups.

In this work, pyrrole-based MIPs were co-polymerized with filamentous bacteriophages and cross-linked by cyclic voltammetry (CV) to form a polypyrrole-phage complex. Thus, we can hypothesize that bacteriophage-derived MIPs provide functional binding cavities to acidic pharmaceutical compounds. Acidic, aromatic, and nucleophilic amino acids were electrochemically polymerized with pyrrole polymers. The MIPs were characterized using QCM-D, scanning electron microscopy (SEM), and UV/VIS spectroscopy, in order to evaluate their binding properties and morphological changes.

## 2. Materials and Methods

### 2.1. Reagents and Materials

The CA (purity, 97%), pyrrole (reagent grade, 98%), benzafibric acid (purity, 98%), diclofenac sodium salt, and sulfamethoxazole were purchased from Sigma-Aldrich (St. Louis, MO, USA). A 10 mM pyrrole solution was prepared in a phosphate buffer (0.1 M NaH_2_PO_4_, sodium dihydrogen phosphate, and 0.1 M Na_2_HPO_4_, sodium hydrogen phosphate) containing 0.1 M KNO_3_ (potassium nitrate), and was stored at 4 °C to prevent oxidation. Deionized water (Di water) from a Millipore Milli-Q purification system was prepared for the solutions. Solutions for polymerization and binding were filtered before use with 0.2 µm polypropylene syringe filters. The stock solutions of benzafibric acid (1 mM), diclofenac (1 mM), sulfamethoxazole (1 mM), and CA (1 mM) were prepared in a 100-mL volumetric flask containing a phosphate buffer (0.1 M NaH_2_PO_4_, sodium dihydrogen phosphate, and 0.1 M Na_2_HPO_4_, sodium hydrogen phosphate) containing 0.1 M KNO_3_ (potassium nitrate), respectively. After being shaken for one hour at room temperature, the stock solutions were stored at 4 °C.

### 2.2. Amplification and Purification of Phage

The *Escherichia coli* K91BluKan (K91BK) strain (kindly provided by Prof. Dr. Georg P. Smith, University of Missouri, Columbia, MO, USA) was used as a host for the amplification of the filamentous bacteriophage particles (phage) [41]. The isolation of the phages from the host cells was performed by the polyethylenglycol/sodium chloride (PEG/NaCl) precipitation method, as previously described [41], after growing the transformed K91BK cells in a Luria broth (LB) supplemented with tetracycline (20 μg/mL) and kanamycin (100 μg/mL) at 37 °C, with vigorous shaking (260 rpm) overnight. The phage supernatants were dialyzed in distilled water to remove the remaining polyethylene glycol (PEG, MW 6000). The samples were dialyzed with a Mw 100 kD cut-off membrane at 120 rpm, stirring overnight at 4 °C (Biotech CE tubing, Rancho Dominguez, CA, USA). The concentration (colony-forming unit per milliliter [cfu/mL]) of the dialyzed samples was measured by UV/VIS. The concentration of the filamentous bacteriophages was determined by phage titration, as previously described [28]. The phage suspensions were stored at 4 °C.

### 2.3. Electrochemical Polymerization of MIP and MIP with Filamentous Bacteriophage (MIP-Phage)

The CV was performed using an electrochemical flow module of a QCM-D system (Q-sense, Västra Frölunda, Sweden). This consists of a working electrode, a counter electrode, and an Ag/AgCl reference electrode. The film deposition was performed with the QCM 401 module, which is a drop-casting electrochemistry system. The determination of the polymer surface and the thickness was performed with field emission scanning electron microscopy (FESEM, Inspect F50, FEI, Hillsboro, OR, USA). The cross-sections were prepared using a diamond cutter (6-539-05, Ogura, Japan) and were sputtered with platinum (E-1045, Hitachi, Tokyo, Japan) for observations of the MIP and MIP-phage. The electropolymerization was followed according to Bianca et al. [42]. In brief, the pyrrole solutions were mixed with CA and phage. Non-imprinted polymer (NIP) (10 mM pyrrole), MIP (10 mM pyrrole and 0.5 mM CA), and MIP-phage (10 mM pyrrole, 0.5 mM CA, and 1.1 × 10^13^ cfu/mL wild-type phage; MIP-phage) were separately placed on a standard 5 MHz AT-cut Au-coated quartz crystal. The configuration was operated using a potentiostat (Autolab PGSTAT 12, Metrohm Autolab, Utrecht, The Netherlands) controlled by the GPES software package (Eco Chemie, Metrohm Autolab, KM Utrecht, The Netherlands). Initially, a phosphate buffer (0.1 M NaH_2_PO_4_ and 0.1 M Na_2_HPO_4_) containing 0.1 M KNO_3_ was pumped through the flow module to remove contaminations and air bubbles. Subsequently, the module was filled with the sample solution and was equilibrated for 5 min. Finally, the MIP was prepared on the Au-coated surface of the quartz crystals through CV of the pyrrole monomer, in the presence of CA or phage in an aqueous phosphate buffer (0.1 M NaH_2_PO_4_ and 0.1 M Na_2_HPO_4_) containing 0.1 M KNO_3_ (pH 7) at 21 °C. The potential was cycled between −200 and +800 mV for 40 cycles, at a scan rate of 100 mV/s. Freshly synthesized polypyrrole—phage films were then rinsed with water and immediately analyzed. After electropolymerization, the CA was extracted with methanol/acetic acid (9:1, *v*/*v*) in a shaker (100 rpm) for 16 h. A standard AT-cut Au-coated quartz crystal was used at the 4.9 MHz resonance frequency. Information about the absorption process is obtained from the changes in the resonance frequency (Δf) and the dissipation factor (ΔD, 1 × 10^−6^) at overtone 7, which represent the harmonic resonances of the base frequency (Figure S1). The CA extraction and rebinding procedure was monitored by QCM-D analysis during several repeated cycles.

### 2.4. Analysis of the Viscoelastic Properties and Topography of the Polypyrrole Matrix

To monitor the immobilization of the polypyrrole–phage films on the Au electrode, we measured the increase in the mass and the viscoelastic properties of the polypyrrole and polypyrrole–phage films using QCM-D analysis. A standard 5 MHz Au-coated quartz crystal was excited at the resonance frequency (Δf, Hz). Information about the adsorption process was obtained from the changes in the dissipation factor (ΔD, 1 × 10^−6^) at overtone number 7 for the crystals. The measurements were performed at a flow rate of 100 µL/min, at 25 °C. The resultant Δf and ΔD were analyzed by the Voigt model using the Qtools software package (Q-sense, Västra Frölunda, Sweden) to extract information on the density, viscosity, shear, and binding mass. To analyze the parallel topography of the polypyrrole surface, electrodeposited films were observed by SEM (FEI Quanta FEG 250, FEI, Hillsboro, OR, USA).

### 2.5. UV/VIS Spectroscopy Measurement

An ultraviolet–visible (UV/VIS) analysis was performed on a solution of 1 mM pyrrole, 1 mM CA, and 1 × 10^12^ cfu/mL bacteriophage by UV/VIS spectroscopy (Ultrospec 3300 pro, Amersham, UK). All of the prepared samples in distilled water were tested over the wavelength range of 200–350 nm. The mixture, prior to electropolymerization, was characterized by UV/VIS spectrophotometry at different intervals to monitor the reaction.

### 2.6. Liquid Chromatography—Tandem Mass Spectrometry Analysis

The CA was quantified in the samples with a high-performance liquid chromatography (HPLC) system (Agilent 1290, Agilent Technologies, Santa Clara, CA, USA) connected to a triple quadrupole mass spectrometer (MS/MS, Agilent 6460, Agilent Technologies, Santa Clara, CA, USA) equipped with electrospray jet stream technology operating in negative ion mode. The operating parameters of the MS were as follows: capillary voltage of 3500 V, nebulizer pressure of 45 psi, drying gas flow of 10 L/min, gas temperature of 300 °C, sheath gas flow of 11 L/min, sheath gas temperature of 350 °C, and nozzle voltage of 1000 V. The separation of the target compound and its internal standard (4-chlorophenylacetic acid, Sigma Aldrich, St. Louis, MO, USA) was performed on a Zorbax rapid-resolution high-definition column (2.1 mm × 50 mm, 1.8 µm particle size, Agilent, Stuttgart, Germany), maintained at 30 °C. To protect the chromatographic column, a C18 guard column (Zorbax Eclipse Plus 2.1 mm × 5 mm, 1.8 µm, Agilent, Stuttgart, Germany) was connected to the column. We used a gradient method with three mobile phases, as follows: Di water containing 0.1% formic acid and 50 mM ammonium acetate (mobile phase A), LC/MS grade acetonitrile (mobile phase B), and LC/MS grade methanol (mobile phase C). The initial conditions were 85% (A), 9% (B), and 6% (C), and this ratio was kept constant for 1.5 min. Phases B and C were then increased to 59% and 39%, respectively, over 4.5 min, and then kept constant for another 1.5 min. All of the conditions of the mobile phases were set back to the initial conditions within 0.5 min. The total running time, including an additional 4 min of a post-run protocol, was 12 min. The flow rate was maintained at 0.4 mL/min, and the sample injection volume was set to 20 µL. The MS analysis in the multiple reaction monitoring (MRM) mode for both CA and its internal standard was carried out using the instrument settings described in Table S2. We accurately monitored the mass of the precursor ions and the transitions to two product ions. The most abundant transition was used for the quantification, whereas the second most abundant was used to confirm the target analyte. These source and fragmentation parameters were optimized for each analyte using 1 mg/L of a single standard dissolved in methanol by passing the same column, described above. A calibration curve for CA was established at five levels ranging from 0.02 to 10.0 µM, with a correlation coefficient R^2^ > 0.99. The Mass Hunter qualitative and quantitative analysis software packages (v.B.06.00, Aligent, CA, USA) were used for data processing.

### 2.7. Selectivity of MIP and MIP-Phage

Three pharmaceuticals were chosen for selectivity test which were benzafibric acid, diclofenac, and sulfamethoxazole. NIP, MIP, and MIP-phage coated Au-electrode were washed with methanol:acetic acid (9:1, *v*:*v*) for 5 min. A binding test was performed at a flow rate of 100 µL/min at 25 °C, and compared with all of the results of NIP, MIP, and MIP-phage with CA, benzafibric acid, diclofenac, and sulfamethoxazole.

### 2.8. Reusability of MIP and MIP-Phage

The CA (1 mM) was adsorbed onto the MIPs in KNO_3_ (100 mM) buffer for 5 min, and subsequently washed with methanol:acetic acid (9:1, *v*:*v*) ,followed by stabilization in KNO_3_ (100 mM) buffer for 10 min. After stabilization, the adsorption of CA and the rebinding procedure were repeated during several experiments.

## 3. Results and Discussion

### 3.1. Electropolymerization of Polypyrrole—Phage Biocomposites

**Figure 1:**
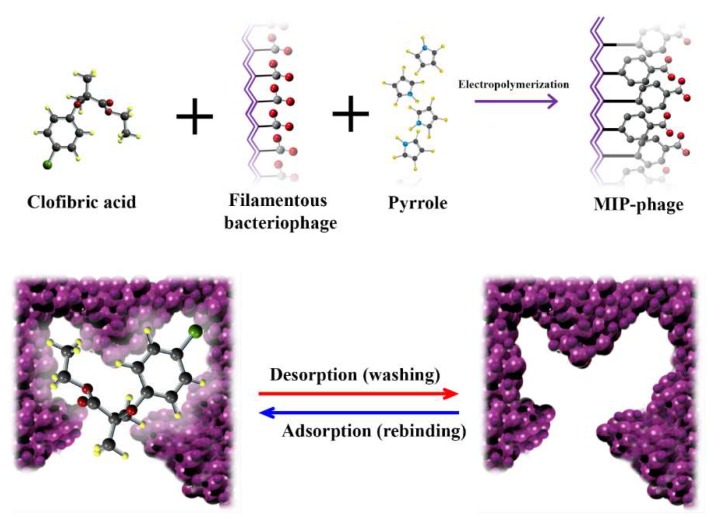
Schematic drawing of the polymerization of the molecularly imprinted polymers (MIPs).

Figure 1 shows a schematic diagram illustrating the construction of the polypyrrole–phage-based biocomposite. In the mixture, complexes form between a filamentous phage and CA, which possess functional amino groups complementary to those on the CA template. Cross-linking polypyrrole monomers are added and the mixture is maintained under electropolymerizing conditions in order to permanently and rigidly fix the spatial arrangement of the functional monomers. Following co-polymerization, the CA is extracted from the polymer matrix, leaving behind cavities whose size, shape, and chemical functionality complement those of the template. These empty cavities can selectively and reversibly rebind molecules similar to the original template. When the pyrrole is mixed with the phage, major pVIII-coated amino acids might be bound to CA, owing to the eleven variable amino acid residues of the C-terminal cytoplasmic domain [25].

**Figure 2:**
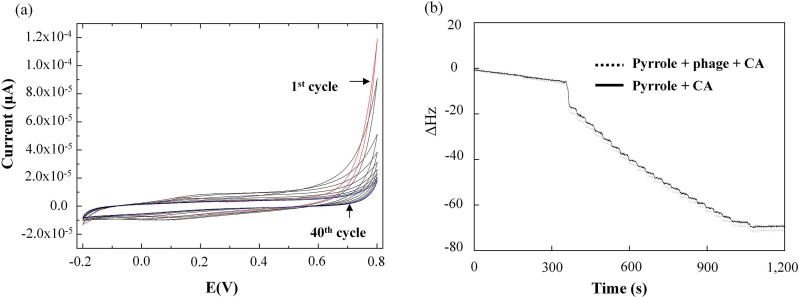
Cyclic voltammograms during preparation of polypyrrole film (**a**) and quartz crystal microbalance with dissipation monitoring (QCM-D) analysis (**b**) during electropolymerization of 10 mM pyrrole containing 0.5 mM clofibric acid (CA) in a phosphate buffer with 100 mM KNO_3_ (solid line) and wt-bacteriophage (1.1 × 10^13^ cfu/mL, dashed line) with 40 cycles between −0.2 and 0.8 V, and a scan rate of 100 mVs^−1^, respectively.

Cyclic voltammograms combined with QCM-D were used to monitor the polypyrrole–phage electropolymerization. Figure 2 shows the accumulated curve obtained after 40 independent measurements. The CV measurement of the redox reaction yielded an estimated stepwise-decreasing plot in the electrochemical module of QCM-D. On the plateau-shaped cyclic voltammograms between −0.2 and 0.8 V, shown in Figure 2a, the peaks due to the oxidation and reduction of the film increase in intensity with the polypyrrole film formation. A broad oxidation peak was observed at a peak potential of 0.2 V, and a reverse reduction peak was seen at a peak potential of 0.1 V. The deposition of the polymer was monitored by QCM-D, where the quartz crystal connected to a potentiostat is also the working electrode. There is no significant difference between the CV curves obtained from pyrrole and pyrrole-phage, indicating that the electron transfer abilities of the phage in the trapped pyrrole are similar to those of the polypyrrole composition (Figure 2b).

### 3.2. UV/VIS Characterization of Pyrrole, Phage, and CA Complexes

**Figure 3:**
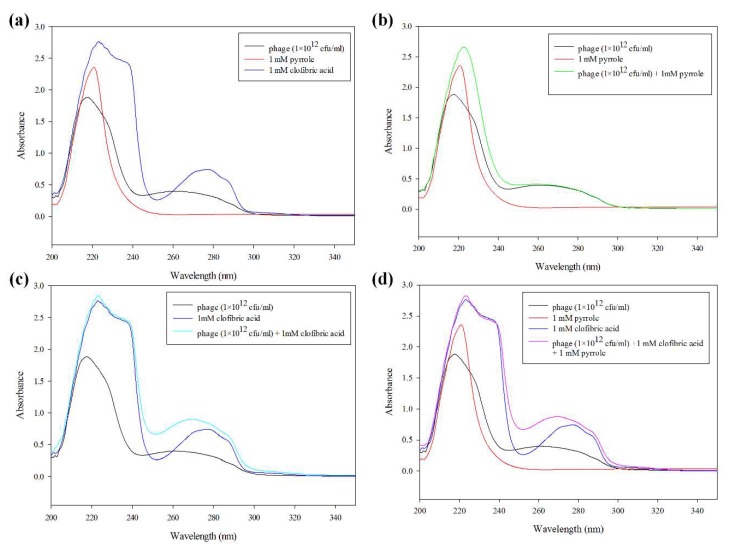
UV/VIS spectra of wt-phage, pyrrole, and CA. All of the samples were prepared in Deionized (Di) water at wt-phage (1 × 10^12^ cfu/mL), pyrrole (1 mM), and CA (1 mM) mixing ratio. phage (black); 1 mM pyrrole (red); 1 mM CA (blue, **a**), phage mixed with pyrrole (green, **b**), phage mixed with CA (cyan, **c**); phage and pyrrole mixed with CA (pink, **d**), respectively.

To evaluate the interaction between py, bacteriophage, and CA, the UV/VIS absorption intensities were measured, as all of the compounds have the aromatic ring structures. In particular, py has a pyridine structure, for which the representative UV/VIS curves are shown in Figure 3a. Previous investigations have reported that the pyridine structure can exhibit the interaction of py–py stacking [28,43,44]. Thus, we acquired a UV-VIS absorption spectra for a mixture of bacteriophage, pyrrole, and CA prior to polymerization. The broad characteristic UV-visible absorption between 200 and 350 nm contains maximum absorption bands at the wavelengths 224, 269, and 275 nm. The filamentous bacteriophages have a characteristic peak at 269 nm [28]. The characteristic peak of the amide NH band appeared at 224 nm, as previously described [45]. As shown in Figure 3a, these correspond to filamentous bacteriophage (wt-phage), pyrrole, and CA solution, respectively. Their characteristic absorption shoulders are clearly noticeable in the curves for all of the mixtures (Figure 3d). Only the mixture of phage and pyrrole, in comparison to the phage solution, exhibited a slightly decreased absorbance peak at 224 nm, in contrast to an increased absorption peak at 269 nm (Figure 3b). It seems that the mixture of phage and pyrrole forms only weak interactions with the amino acid residues of the phage in the form of ionic interactions. Based on computer simulations, Rahim Ghadari reported that the aromatic amino acids Phe, Trp, and Tyr have the most favorable binding affinities toward pyrrole-like moieties, owing to stabilization by π–π stacking interactions and hydrogen bonding. Among the three amino acids, Trp appears to be the most effective π–hydrogen bond acceptor in the proteins [46]. As described in Table S1, the filamentous bacteriophage presents not only filamentous folded structures on its surface, but also an aromatic ring of amino acids (e.g., pVIII Phe [F] 4 amino acid [aa], Tyr (Y) 2 aa, and Trp [W] 1 aa; pIII F 22 aa, Y 21 aa, and W 4 aa; pVI F 10 aa, Y 4 aa, and W 1 aa; pIX F 3 aa, Y 2 aa, and W 1 aa; and pVIII F 2 aa, Y 1 aa). Thus, we speculate that a small amount of the change in *λ_max_* is due to conformational changes in Phe, Trp, and Try in the presence of the benzene ring of pyrrole.

### 3.3. SEM Analysis of Polypyrrole Matrix Topography

**Figure 4:**
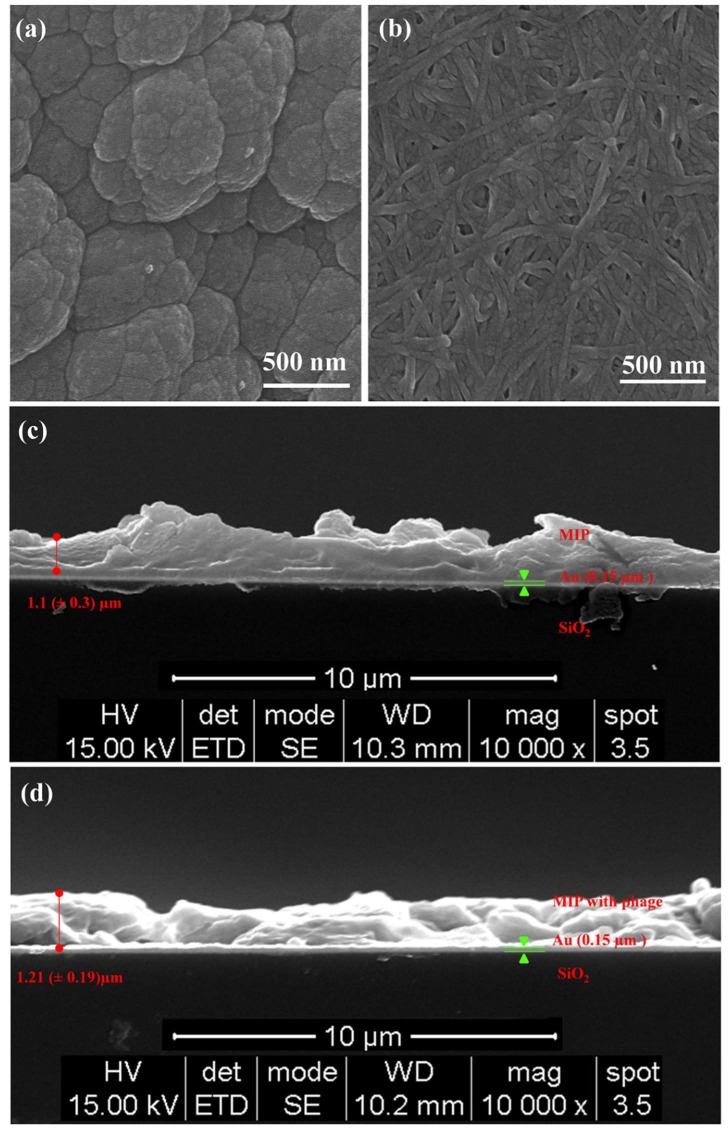
Field emission scanning electron microscopy (FESEM) images illustrating the surface morphologies, thickness, and roughness. The polymerization of pyrrole (MIP) top-view (**a**), cross-sectional view (**c**), pyrrole with wt-phage (MIP-phage) top-view (**b**), and cross-sectional view (**d**).

The SEM analyses shown in Figure 4 characterize the polypyrrole and polypyrrole–phage films on an Au surface. Figure 4a shows the typical sparse grains of a cauliflower-like polypyrrole matrix [47]. It demonstrates that the matrix was successfully prepared using the electropolymerization method. In contrast to this, the surface morphology of the MIP-phage exhibited cross-linked structures in the patch-like surfaces, as shown in Figure 4b. In this figure, large agglomerates of phage bundles, smaller clusters of phages, and individual phages in the polypyrrole layer were observed. The characteristic features of the filamentous bacteriophages were approximately 6.6 nm wide and 100 nm long [26]. However, the conjugation of pyrrole and wt-phages resulted in the encapsulation of wt-phages and showed a thickness of approximately 100 nm, indicating that the phages were successfully incorporated into the polypyrrole biocomposites. The MIP with filamentous bacteriophage complex may allow for further modification of the structure and roughness following polymerization. After the desorption process of the CA, we measured the average thickness values using FESEM, as shown in Figure 4. From these figures, the thicknesses of MIP (c) and MIP with wt-phase (d) were calculated as 1.1 (±0.3) µm and 1.21 (±0.19) µm on the Au-coated electrode using the Image J software package (National Institutes of Health, Bethesda, MD, USA), respectively. This result revealed that the thickness was not influenced by the biochemical properties, but that different structures and roughness could be generated by the filamentous bacteriophage. This new morphology could lead to a significant increase in the size of the repeating structure and binding cavities of the polymer networks, because the cross-linked structures provide a larger number of porosity sites for CA.

### 3.4. Interaction of CA with the Polypyrrole Matrix on a Microbalance Sensor

**Figure 5:**
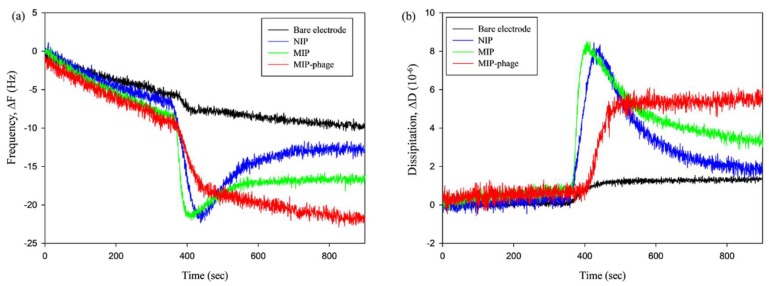
QCM adsorption analyses of bare electrode (black line), nonimprinted polymer (NIP) (blue line), MIP (green line), and MIP-phages (4 × 10^12^ cfu/mL, red line); frequency (**a**) and dissipation shift (**b**).

QCM-D experiments were performed to investigate the binding affinity between CA and different types of MIPs in more detail. The frequency shift (Figure 5a) showed a similar pattern to the dissipation shift (Figure 5b) in the QCM-D analysis. The black (bare electrode) and blue (NIP) lines represent a control experiment for CA adsorption. Injecting a 1 mM CA solution into the flow channel led to strong increases in the frequency and dissipation signals. The MIP and NIP showed an initial frequency increase of approximately −20.84 Hz (∆f), and 8.13 × 10^−6^ (∆D) upon the injection of CA for 200 s. All of the lines were saturated after 400 s. In particular, the NIP closely approached the bare electrode frequency levels and, then, all of the MIPs were saturated at different levels. These results indicate that the CA was initially nonspecifically bound to the MIP and NIP surfaces. The resulting ∆f/∆D values for MIP-phage (∆f [−22.65] Hz/∆D 5.36 × 10^−6^) are much higher than those for MIP alone (∆f [−16.35] Hz/∆D 3.20 × 10^−6^). This suggests that MIP-phage is more effective and has a higher affinity to CA than MIP alone. For Figure 1 and Figure 4, as well as these results, we carefully assumed that the bacteriophages provide not only a size extension, but also a number of effective functional cavities that use NH_3_^+^, COO^−^, and aromatic rings to MIPs. For this reason, MIP-phage composes a binding pocket and makes the cavity for CA, which probably shows interactions such as hydrophobic contacts and π–π stacking between the aromatic rings [48]. We included the QCM adsorption analysis graph of all of the overtones (Figures S1 and S2). Figure S1 below shows that all of the overtones have a similar pattern. From the comparison of all of the overtones, we found that the frequency shift tended to be highly sensitive at the low overtone numbers. The low overtones (*n* = 1, 3, and 5) needed more time for stabilization than the higher overtones. Although all of the overtones showed a similar pattern, the high overtones showed more noise (*n* = 9, 11, and 13) (Figure S2). Similar trends were reported previously [49,50]. Thus, we selected overtone number 7.

### 3.5. Binding Efficiency and Capacity of CA to Polypyrrole Matrix on Microbalance Sensor

**Figure 6:**
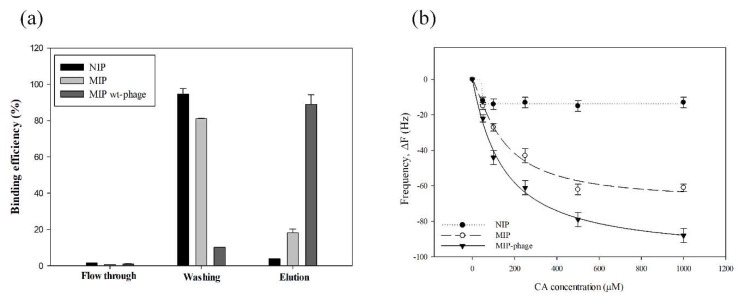
Adsorption and binding capacity of NIP, MIP, and MIP-phage. The CA binding affinity from flow through, washing, and elution of the samples by LC-mass spectrometer (MS)/MS analysis (**a**). CA removal from NIP and MIPs was carried out in buffer with 10% acetic acid in the elution step. The changes in frequency from 0 to 1 mM concentration of CA (**b**). Data were analyzed statistically by ANOVA at a *p* < 0.05 confidence interval (*n* = 3).

In order to confirm specific interactions of the NIP and different types of MIP, a concentration-based frequency change and a recovery experiment were conducted. The surface was washed with Di water after the adsorption of CA (1 mM). For the evaluation of the recovery on the CA-imprinted polymer, the recovery potential of the polymers was tested by LC-MS/MS with quantification of CA from the flow through (PBS), washing of (Di water), and elution of (MeOH, acetic acid 9:1, *v*:*v*) the media samples. The results for the CA recovery (%) are shown in Figure 6a, namely: NIP (1.53%), MIP (0.65%), and MIP-phage (0.91%) in the flow through sample; NIP (94.65%), MIP (81.13%), and MIP-phage (10.15%) in the washing sample; and NIP (3.82%), MIP (18.21%), and MIP-phage (88.94%) in the elution sample. Figure 6b shows the frequency responses for different concentrations of CA ranging from 50 μm to 1 mM. 

In order to calculate binding affinities, we used the Hill regression model, as shown in Table 1. Based on the regression model parameters in Table 2, the binding affinities were calculated by Michaelis–Menten kinetics. The dissociation constant (K_D_, µM·L^−1^) was characterized by the maximum absorption of CA (*V_max_*, Hz [∆f]). The Michaelis constant (K_m_, µM·L^−1^) was the concentration at which the reaction rate was half of *V_max_*. *K_D_*, and was determined as follows:

(1) $$K_{D}=V_{max}\left[\mathrm{CA}\right]\;\cdot \;{K_{m}}^{-1}$$

The linear regression equation of NIP is y = −13.7500 × x^24.6453^ × (46.2427^24.6453^ + x^24.6453^)^−1^ (r^2^ = 0.9822), *K_m_* = 3.95 × 10^39^, *V_max_* = −13.75, K_D_ = 3.48 × 10^−39^; MIP is y = −68.7239 × x^1.2715^ × (142.6995^1.2715^ + x^1.2715^)^−1^ (r^2^ = 0.9874), *K_m_* = 354.63, *V_max_* = −63.392, and *K_D_* = −1.79 × 10^−1^; and the MIP-phage is y = −99.0498 × x^1.0592^ × (143.4342^1.0592^ + x^1.0592^)^−1^ (r^2^ = 0.9950), *K_m_* = 145.23, *V_max_* = −87.821, and *K_D_* = −6.05 × 10^−1^. A previous investigation reported that divinylbenzene 80-based MIP had a limit of detection of 35.03 µM of CA in water samples, using a HPLC analysis system [19]. As shown at Figure 6b, the on/off sensing efficiency can be clarified for MIP-phage from 50 µM CA and for MIP from 100 µM CA. The MIP-phage in this study has a higher detection limit than the MIP for CA absorption. These systems have advantages for efficient live on/off sensing of targets and high sensitivities, which are similar to the HPLC detection limits. The reactive frequency can be clearly distinguished from the binding signals above 50 μM of CA. In the environment, CA is mostly found in concentrations below 100 μΜ. Emudianughe et al. showed that after doses of 500–1500 mg CA, human urine contained the following concentrations of CA: 46.5–223.6 µM at 0–24 h and 11.6–28.0 µM at 24–48 h [51]. In waste water, CA exists in a wide range, from pM to nΜ concentration [52,53]. Thus, we assumed that MIP-phage can probably be used to diagnose CA during the early stages (0–24 h after doses of CA) in urine samples. These systems cannot be adapted to the environmental diagnosis field, because of low concentrations of pharmaceutic residue. However, these systems can be applied in the water purification field, because our results show that MIPs are selective and reusable. Furthermore, previous research showed that MIPs were stable over wide ranges of temperature, pH, and humidity [54]. The interaction of CA with the MIP-phage biocomposite further confirmed that CA has a higher affinity for MIP-phage than for MIP and NIP composites. This observation suggests that the components of the wt-phage, such as NH_3_^+^, COO^−^, and aromatic rings, could form complexes with CA in the polymer matrix. This may yield a higher CA binding affinity and sensitivity compared to the polypyrrole composites. In particular, we previously reported that the selective adsorption of CA compared to caffeine was achieved [42]. Similar to our finding for CA absorption to MIP, we hypothesize that phages decorated with amino acids can improve the binding affinity to CA. Based on our study, it appears as if the MIP and wt-phage complex provides patch-like structures that can yield different thickness and roughness compared to MIP and NIP. Thus, we assumed that multiple interactions contributed to the absorption of CA, which were based on the different physicochemical properties of the functional amino acids in the binding cavities. 

### 3.6. Binding Selectivity of NIP, MIP, and MIP-Phage

**Figure 7:**
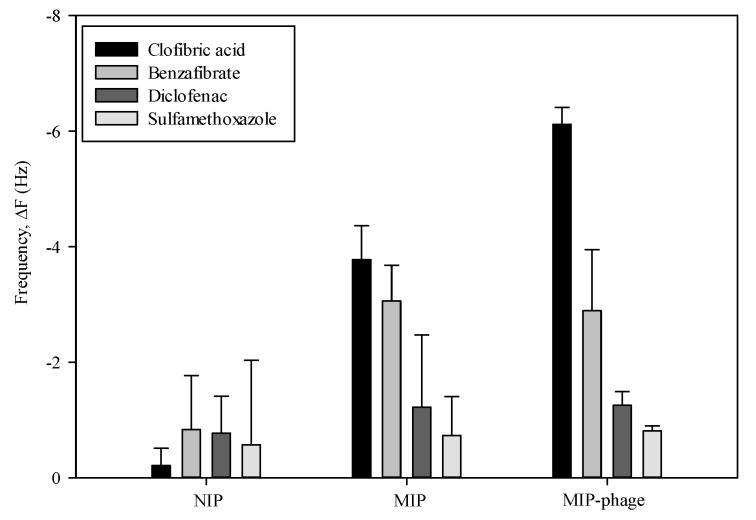
Binding selectivity of NIP, MIP, and MIP-phage. The changes of frequency from 1 mM of CA, benzafibrate, diclofenac, and sulfamethoxazole on MIP-phage, MIP, and NIP, respectively (*n* = 3).

CA absorption experiments were carried out in order to examine the binding selectivity of the NIP, MIP, and MIP-phage. The responses of the frequency change were tested by three pharmaceutical compounds. As shown in Figure 7, the MIP exhibited a binding affinity for the CA (−3.78 ± 0.58 Hz) and benzafibric acid (−3.06 ± 0.62 Hz) molecule, whereas, the adsorption efficiency of diclofenac (−1.22 ± 1.25 Hz) and sulfamethoxazol (−0.73 ± 0.67 Hz) was much lower than that of the CA molecules. Quite interestingly, the MIP-phage showed a significantly higher binding selectivity for CA (−6.12 ± 0.29 Hz) than other compounds, which were benzafibrate (−2.89 ± 1.06 Hz), diclofenac (−1.26 ± 0.23 Hz), and sulfamethoxazol (−0.81 ± 0.08 Hz), respectively. The results showed that the MIP-phage had a higher CA molecular recognition selectivity to its template. The adsorption efficiency of NIP for CA was lower than that of MIP; on the other hand, their adsorption capacities for the benzafibric acid were similar to CA due to the similar structural analog to the template molecules.

### 3.7. Reusability of MIP and MIP-Phage

**Figure 8:**
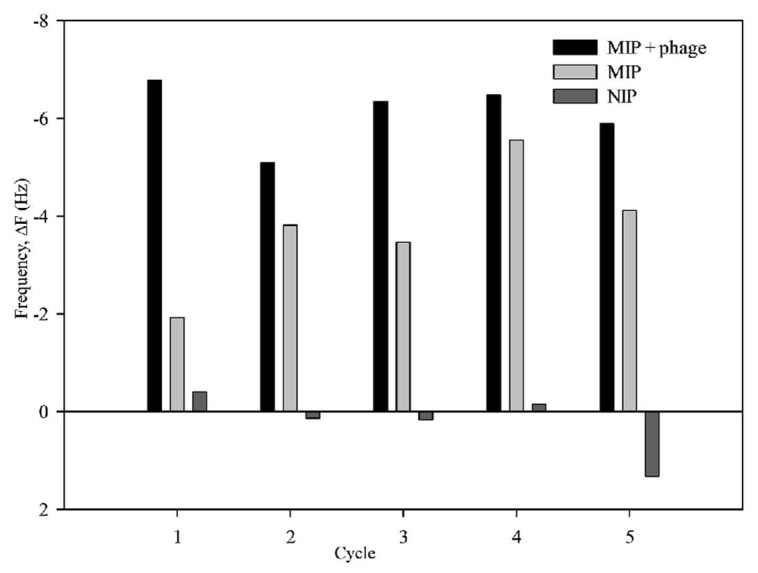
MIPs regeneration and reusability. Frequency changes in five repeated runs indicate adsorption and desorption of CA on MIP-phage, MIP, and NIP, respectively.

The reusability of MIP was tested by performing five sequential adsorption and desorption regeneration cycles (Figure 8). The captured CA on MIP-phage was elutriated and washed using a methanol:acetic acid (9:1, *v*:*v*) mixture. As there was no obvious decrease in the adsorption efficiency for CA, this proves that the MIP can be used for at least five cycles, and it has a certain regeneration adsorption efficiency. To evaluate the robustness, we calculated the average absorption frequencies, which were −6.12 ± 0.29 Hz (MIP-phage), −3.78 ± 0.58 Hz (MIP), and 0.21 ± 0.30 Hz (NIP). A comparison of these results shows that the MIP-phage was more stable and sensitive than MIP. From these results, we inferred that bacteriophages supported the robustness of MIPs, because filamentous bacteriophage had linear structures and were consisted of a large number of protein complexes. It was likely that the filamentous bacteriophages supported not only the functional cavities, but also the robustness of the polymer matrix, owing to the scaffold effects. All of these are signs of good reusability. Moreover, the five rebinding cycles of CA onto the polypyrrole–phage composites did not influence the sensitivity of the QCM analysis. This suggests that detecting acidic pharmaceutic compounds on a microbalance sensor is a highly effective methodology with sufficiently high sensitivity, affinity, and long-term stability.

The sensitivity of the imprinted polymer and its imprinting specificity were tested with MIP, NIP, and MIP-phage. The MIP-phage showed a higher response to CA than NIP and MIP. This high binding affinity might have resulted from the structure and functionality changes of MIP-phage.

Filamentous bacteriophages are decorated with polar and nonpolar amino acid side chains, which may explain the effective adsorption ability. To gain a better understanding of the performance of the MIP-phage sensor, we will try to test different fibrate drugs and real waste water samples with MIP-phage on the QCM sensors in further studies.

## 4. Conclusions

A novel MIP was successfully synthesized with pyrrole, filamentous bacteriophage, and CA by electropolymerization. The surface morphology was changed from a cauliflower-like polypyrrole matrix to cross-linked structures on patch-like surfaces. The thickness was increased from 1.1 (±0.3) µm MIP to 1.21 (±0.19) µm for the MIP-wt-phage. We inferred that the bacteriophages provide not only a size extension, but also a number of effective functional cavities that use NH_3_^+^, COO^−^, and aromatic rings to MIP. For this reason, MIP-phage composes a binding pocket and makes the cavity for CA, which probably shows interactions such as hydrophobic contacts and π–π stacking between the aromatic rings. MIP-phage can be reused for more than five times without influencing the sensitivity. The new method appears to be a promising, unique, and versatile technique with a high sensitivity and efficient reproducibility, which should have many applications in the fields of sensors, electronics, and biomedical engineering. To gain a better understanding of the performance of the MIP-phage sensor, we will try to test different fibrate drugs and real waste water samples with MIP-phage on the QCM sensors in further studies.

## Figures and Tables

**Table 1:** Regression models employed in describing the binding efficiency ratio and concentration—dependent frequency curves in this study.

Regression Model	Function
Hill (H)	$F\left(c\right)=\frac{\alpha c^{\beta }}{\gamma^{\beta }+c^{\beta }}$
Binding efficiency (%)	$B\left(\%\right)=100-\left(\left(\left(F+W+E\right)\cdot 3^{-1}-N\right)\cdot {\left(F+W+E\right)}^{-1}\right)\cdot 3^{-1}\times 100$

Note: *F*(c)—the frequency elicited at concentration *c*; *α*, *β*, and *γ*—parameters of regression models (corresponding statistical estimates); *B*(%)—binding efficiency; *F*—flow through; *W*—washing, *E*—elution; *N*—flow through or washing or elution.

**Table 2:** Parameters of regression models for frequency curves on NIP nonimprinted polymer (NIP), molecularly imprinted polymers (MIP), and MIP-phage of clofibric acid (CA).

Imprinted Polymers	RM ^1^	R ^2^	Model Parameter		
			A ^2^	Β ^3^	Γ ^4^
NIP	H	0.982	−13.7500	24.6453	46.2427
MIP	H	0.987	−68.7239	1.2715	142.6995
MIP-phage	H	0.995	−99.0498	1.0592	143.4342

^1^ Regression models (H—Hill); ^2^ height; ^3^ slope; ^4^ center point.

## Supplementary Materials

The following are available online at http://www.mdpi.com/2073-4360/10/9/974/s1.
