# Supplementary material for: Detection of Acidic Pharmaceutical Compounds Using Virus-Based Molecularly Imprinted Polymers
Source: Polymers (Basel). 2018 Sep 1;10(9):974. doi: 10.3390/polym10090974 (PMC6403656; doi:10.3390/polym10090974)
Supplement: Supplementary file 1 [file polymers-10-00974-s001.pdf]

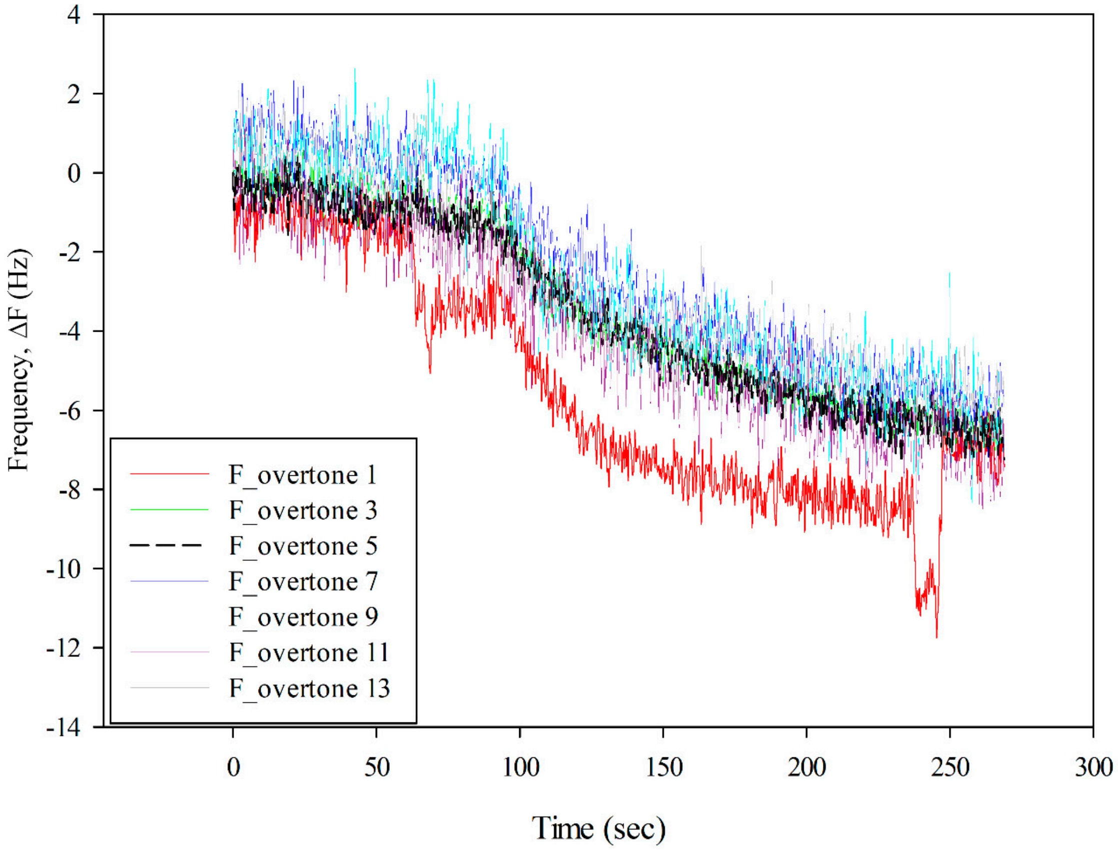

Figure S1. QCM adsorption analysis of seven overtones (1, 3, 5, 7, 9, 11, 13) based on MIP with bacteriophage.

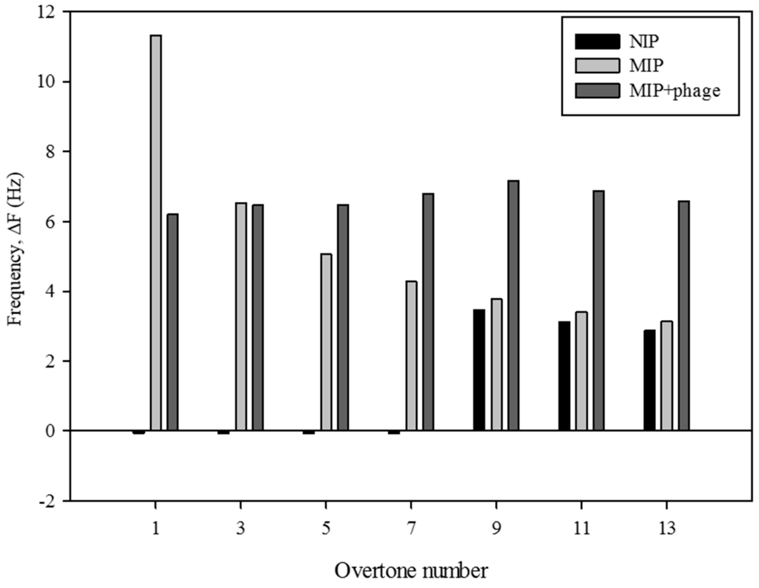

Figure S2. Frequency changes of MIP, NIP, and MIP with phage at seven overtones.

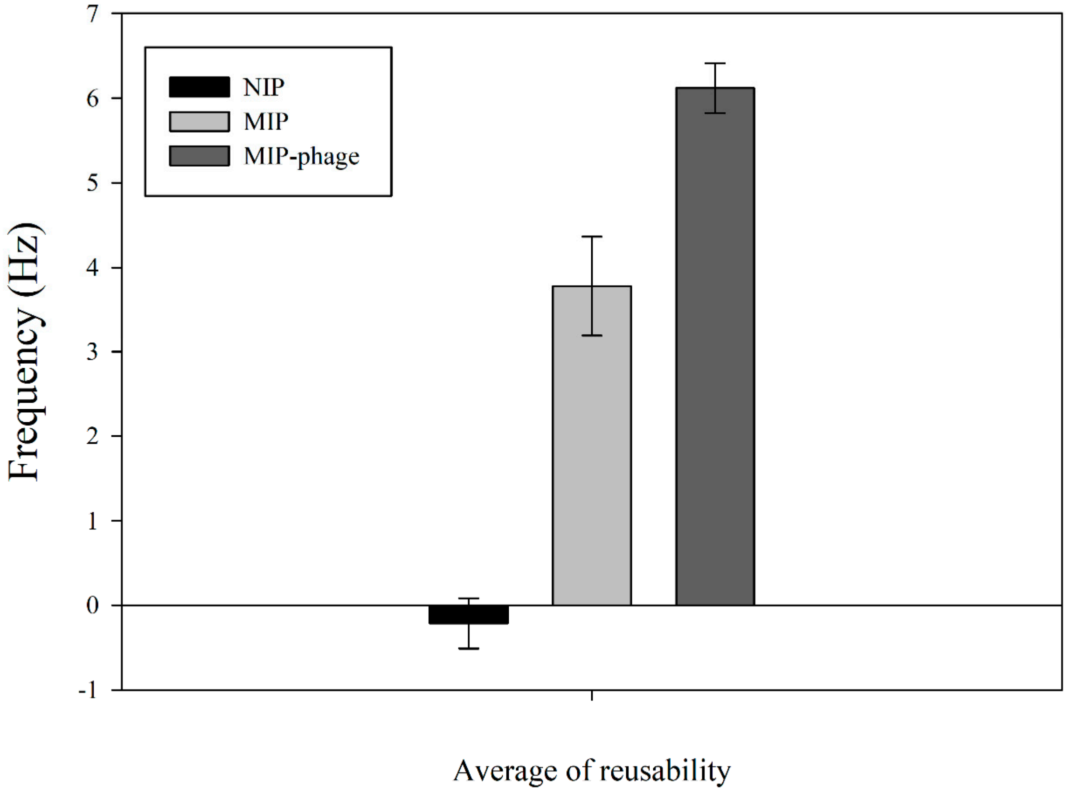

Figure S3. Average of absorption frequency.

Table S1. M13 filamentous bacteriophage protein information (accession number NC_003287 from NCBI)

Major coat protein (pVIII) – 1301 – 1522

MKKSLVLKASVAVATLVPMLSFAAEGDDPAKAAFNSLQASATEYIGYAWAMVVVIVGATIGIKLFKKFTSKAS

Minor coat protein (pIII) 1579–2853

MKKLLFAIPLVVPFYSHSAETVESCLAKPHTENSFTNVWKDDKTLDRYANYEGCLWNATGVVVCTGDETQCYGTWV
PIGLAIPENEGGGSEGGGSEGGGSEGGGTKPPEYGDPIPGYTYINPLDGTYPPGTEQNPANPNPSLEESQPLNTFMFQN
NRFRNRQGALTVYTGTVTQGTDPVKTYYQYTPVSSKAMYDAYWNGKFDCAFHSGFNEDPFVCEYQGQSSDLPQPPV
NAGGGSGGGSGGGSEGGGSEGGGSEGGGSEGGGSGGGSGSGDFDYEKMANANKGAMTENADENALQSDAKKLDS
VATDYGAAIDGFIGDVSGLANGNGATGDFAGSNSQMAQVGDGDNSPLMNNFRQYLPSLPQSVECRPFVFSAGKPYE
FSIDCDKINLFRGVFAFLLYVAFMYVFSTFANILRNKES

Minor coat protein (pVI) 2856 – 3194

MPVLLGIPLLLRFLGFLLVTLFGYLLTFLKKGFGKIAIAISLFLALIIGLNSILVGYLSDISAQLPSDFVQGVQLILPSNALPC
FYVILSVKAAIFIFDVKQKIVSYLDWDK

Minor coat protein (pIX) 1206 – 1304

MSVLVYSFASFVLGWCLRSGITYFTRLMETSS

Minor coat protein (pVII) 1108 – 1209

MEQVADFDTIYQAMIQISVVLCFALGIIAGGQR

Table S2. Negative mode multiple reactions monitoring (MRM) transitions selected for target analyte, CA, and its internal standard, 4-chlorophenylacetic acid, with fragment voltage (FV) and collision energy (CE)

Compound	Mass transition (m/z)	Fragmentor, eV	CE, eV	Usage
Clofibric acid (CA)	213 → 127	68	9	Quantitation
	213 → 85	68	5	Confirmation
4-chlorophenylacetic acid	169 → 125	56	5	Quantitation
	169 → 35	56	13	Confirmation
